# A pilot study of a modification EAT-26 questionnaire for screening pathological eating behavior in competitive athletes

**DOI:** 10.3389/fpsyg.2023.1166129

**Published:** 2023-06-02

**Authors:** Daniela Stackeová, Tereza Barešová, Barbora Přibylová

**Affiliations:** College of Physical Education and Sport Palestra, Prague, Czechia

**Keywords:** EAT-26, Eating Attitudes Test, pathological eating behavior, eating disorders in athletes, anorexia athletica

## Abstract

This study deals with pathological forms of eating behavior and disorders of athletes with the aim to verify a newly created questionnaire method focused on their screening. First, a detailed analysis of one of the most frequently used methods, EAT-26, was carried out, which was subsequently reworked into a newly created questionnaire that should meet the criteria for application to a group of competitive athletes. This new questionnaire was then verified on a group of athletes in risky sports disciplines. It was distributed among athletes of aesthetic sports, specifically among aerobics (gymnastic, sport, and fitness), gymnastics (modern and sport), professional dance, figure skating, and bodybuilding/fitness (classic bodybuilding, bikini fitness, and men’s physique). In total, 100 respondents, 79 women and 21 men, with 20 persons from each sport branch, aged 16–26, participated in the research. The main results of the research investigation were determined using factor analysis, which yielded positive results. Five strong factors (dietary control, body weight control, training obsession, appetite, and calorie counting) were identified, which can be defined as common and well-known characteristics in the eating and training regime of competitive athletes. At the same time, the factors found can be defined as essential factors influencing the emergence of disturbed eating behavior or the later development of an eating disorder. Compared to the original EAT-26, the point score was adjusted, and its critical value was determined at 57 points. Of the respondents, 33%, i.e., 33 out of a possible 100, achieved this value or above. Respondents with a point score of 57 and above were found in every sport tested. Of the 33 respondents reaching this point limit, 6% were in aerobics, 24% in gymnastics, 15% in professional dance, 27% in figure skating, and 27% in bodybuilding/fitness. Respondents from the bodybuilding and fitness sports sector achieved the highest number of points on average and those were the only ones who exceeded the threshold of 57 points on average. The results of the work correspond with the opinion of many experts that the sports environment is ideal for covering up disturbed eating behavior or eating disorders, and diagnosis in this environment is difficult.

## 1. Introduction

We see the topic of pathological eating behavior and psychogenic eating disorders in athletes as extremely relevant. Their incidence is probably much higher than assumed because, among other issues, we lack effective diagnostic tools.

Psychogenic eating disorders in athletes and their causes and manifestations are not identical to their causes and manifestations in the general population. They are far more connected to athletic performance and success in sports than body appearance and body weight (Byrne and McLean, 2001). Contemporary sport at the top-level places extreme demands on athletes in all aspects, including nutrition, and this is all the more relevant in aesthetic sports or in sports in which body weight plays a role within the division into weight categories or as an advantage when performing the sport itself. In some sports, there are fundamental changes in body weight during competitive and non-competitive periods.

For pathological eating behavior in athletes, the term sports anorexia (anorexia athletica) is commonly used. This is a condition described in athletes who restrict energy intake, engage in excessive to extreme exercise, or engage in both, in order to achieve or maintain a low body weight as a way to maximize their athletic performance (Dunford and Doyle, 2007).

Plowman and Smith (2007) states the following diagnostic criteria for anorexia athletica: body weight loss (at least 5% below normal for given age and height), absence of any other health problems that would explain the body weight loss, excessive fear of being overweight, energy intake below 1,200 kcal per day, delayed puberty, amenorrhea or oligomenorrhoea, consumption of foods with low energy content, gastrointestinal problems, disturbed perception of one’s own body, excessive to extreme exercise, and compulsive need for exercise beyond the training regime.

Personal risk factors for the emergence of pathological eating behavior in athletes may include those that are described as typical for athletes, namely competitiveness, determination (motivation), and perfectionism, which is most often mentioned in various works (Dosil, 2008; Bean, 2013).

Bean (2013) describes the factors influencing the development of eating disorders in athletes as the demands of the given sport, pressure from the coach or teammates, social and media pressure, and pressure from the family environment. Dosil (2008) lists a wider spectrum, namely weight restrictions in sports, referee criteria, physical demands of sports that require an extremely low percentage of body fat, pressure from the coach, and pressure from teammates. A similar list is given by Berning and Steen (2005): early specialization in training, diets and cycles, traumatic events, the influence of coaches, and the rules of the given sport discipline.

There are a number of methods for diagnosing disordered eating behavior and eating disorders. The format of a structured interview is usually seen as the most effective method. A structured diary format also offers objective assessment. Many scientists and researchers use less time-consuming questionnaires for their studies (Fichter and Quadflieg, 2000).

The Eating Attitude Test by Garner and Garfinkel is probably the most widely used standardized measuring gauge of symptoms characteristic of eating disorders. The original version of the test is the EAT-40 version, which was first published in 1979 and was used in one of the first studies, which focused on examining sociocultural factors in the development and maintenance of eating disorders. Based on this version of the test, a new and more advanced version was created, which was called the EAT-26. Since then, the test has been translated into many languages and used in hundreds of studies. The original publication of the test (1979) and the subsequent publication describing the refinement of the test (1982) are ranked 3rd and 4th, respectively, on the list of the 10 most cited articles in the history of the magazine of Psychological Medicine. Founded more than 40 years ago, this journal has allowed the Eating Attitudes Test to make a huge impact in the field of eating disorders (Garner, 2017). The Eating Attitudes Test published in 1979 by Garner and Garfinkel is also found by Nathan and Allison (1998) to be the most widely used method for assessing eating disorders. But it is very significant that it cannot distinguish between anorexia nervosa and bulimia nervosa.

Screening for eating disorders is based on the premise that early recognition of the disorders can lead to earlier treatment and thereby reduce serious physical and psychological complications or, in the worst cases, even death. The test should be part of the so-called two-step screening process and should be the first step. The second step is usually determined according to the given methodology related to the final test score. According to this methodology, a respondent with a point score above 20 points should be invited to an interview with a qualified expert (EAT-26, 2017).

The Eating Attitudes Test is based on the following three criteria (EAT-26, 2017):

- *The total score based on answers to EAT test questions.* A score of 20 points or higher indicates an increased risk of one of the eating disorders. It points to an individual’s increased interest in eating and body weight, or disturbed eating behavior itself.
- *Answers to questions concerning the behavior associated with eating and weight loss.* If the respondent answered yes to any of the questions regarding disordered behavior, they should seek a qualified mental health professional who specializes in dealing with eating disorders.
- *Body mass index (BMI) calculated according to height and body weight.* The Eating Attitudes Test contains specific questions about body height, body weight, and gender, the answers to which can be used to calculate body mass index (BMI).

The test is available in a long 40-item version and in a shortened 26-item version, both versions rated on a six-point Likert scale (1 = never, 6 = always). The questions are closed, and the respondent can choose from six answers: always, very often, often, sometimes, rarely, and never. In the evaluation, each answer is assigned a certain number of points. For questions 1–25, they are evaluated as follows: always = 3 points, very often = 2 points, often = 1 point, sometimes, rarely, and never = 0 points. Question 26 is evaluated in the opposite way: always, very often, often = 0 points, sometimes = 1 point, rarely = 2 points, never = 3 points. The resulting sum of points describes the individual’s eating behavior, with a score of 20 and above defined as disordered eating behavior. Dosil (2008) states that the Eating Attitudes Test focuses on three main factors, namely dieting behavior, bulimia and increased interest in food and oral control. It consists of questions about avoiding high-calorie foods, thoughts or anxiety about food and its ingredients, self-control in the area of food intake, and questions about the perception of pressure from others regarding body weight and weight gain/loss.

At this point, we also present a clear table of diagnostic questionnaire methods focused on pathological eating behavior and eating disorders, the comparison of which was used when selecting the method for our study (Table 1).

**Table 1:** Overview table of diagnostic questionnaire methods.

Method name	Year of publishing	Author	Specialization	Length	Advantages	Disadvantages
EDE	1987	Cooper and Fairburn	The psychopathology of eating disorders	–	Interviewee and interviewer interaction	Time-consuming, necessity of trained professional staff
EDDS	2000	Stice et al.	Symptoms of anorexia nervosa, bulimia nervosa and binge eating disorder	22 items	Strong validity and reliability, easy applicability and evaluation	It does not reflect the fact that behavioral symptoms are much less consistent than cognitive symptoms
QEED	1997	Mintz et al.	Assessment of presence of anorexia nervosa, bulimia nervosa and 4 types of EDNOS	50 items	Fast filling, easy evaluation, high sensitivity	Choice of answers only yes/no
SIAB	1990	Fichter et al.	Assessment of presence of anorexia nervosa, bulimia nervosa	87 items	Can be used to derive diagnoses	Time-consuming, does not reflect changes in DSM-5 criteria, necessity of professional training of the staff
BULIT-R	1991	Smith and Thelen	Symptoms of bulimia nervosa	36 items	Easy to apply, takes 10 min to complete, good validity and reliability	Evaluation (possibility of false negative results)
EAT	1979	Garner and Garfinkel	Symptoms characteristic of eating disorders	26 items/40 items	Multifunctionality (evaluates a range of attitudes toward food and of eating behavior, identification of individuals at risk, evaluation of treatment), can really distinguish between disordered eating behavior	Cannot distinguish between anorexia nervosa and bulimia nervosa, not applicable for children under 15 (see children’s version)
EDE-Q	1994	Faiburn and Beglin	Duration and frequency of eating disorder symptoms	36 items	Good validity and reliability	It is not possible to clarify and make rating judgments about more complex and subjective concepts, it is not possible to assess the complex behavior of an individual
EDI	1983	Garner et al.	Psychometric peculiarities and symptoms related to the development and progress of eating disorders	64 items	Translation into many foreign languages, widely used in the sports environment	Cannot be used separately, the contents of some sub-groups does not relate to eating and body weight, but are general psychopathological scales, does not differentiate between eating disorders and psychiatric patients, use in men
SCOFF	1999	Morgan et al.	Basic characteristics of anorexia nervosa and bulimia nervosa	5 items	High sensitivity, memoizable, good validity and reliability	Very short, does not serve as a diagnostic method - expert evaluation is required

## 2. Objective

The main objective of our study was the creation of a method that will be applicable to competitive athletes, who are a highly specific group, without completely creating a new method, but instead modifying the most used questionnaire method EAT-26. The created screening method should be able to recognize pathological forms of eating behavior or eating disorders in athletes. The main difference from the previously developed screening and diagnostic methods intended for athletes and non- should be the inclusion of all important aspects of the athlete’s life in the questionnaire (with regards to the type of sport, type of competition, training regimen, and diet regimen).

The opinion that the current form of the EAT-26 is not ideal for screening eating disorders in athletes is shared by Martínez Rodríguez et al. (2015), who in his study focused on pathological eating behavior in contact sport athletes. On the contrary, Garfinkel and Newman (2001) in their article reflecting on the EAT-26 test’s 25 years of existence, report without doubt on the suitability of this test for athletes and emphasize that the most vulnerable group, as far as eating disorders are concerned, are top-level and professional athletes.

## 3. Characteristics of the research group

The research was conducted on a group of competitive athletes. In order to include a respondent in the research group, the presence of three basic factors was necessary:

- Partakes in a specific training program.
- Partakes in a specific eating program (none of the athletes were currently following a restrictive diet aimed to athletes primarily reduce body weight due to competition weight categories, these were athletes in disciplines where a long-term dietary modification focused on body composition is necessary).
- An active status of an athlete—participation in amateur level competitions/races.

The questionnaire was distributed among athletes of aesthetic sports, namely aerobics (gymnastics, sports, and fitness), gymnastics (modern and sports), professional dance, figure skating, and bodybuilding/fitness (classic bodybuilding, bikini fitness, and men’s physique).

**Figure 1:**
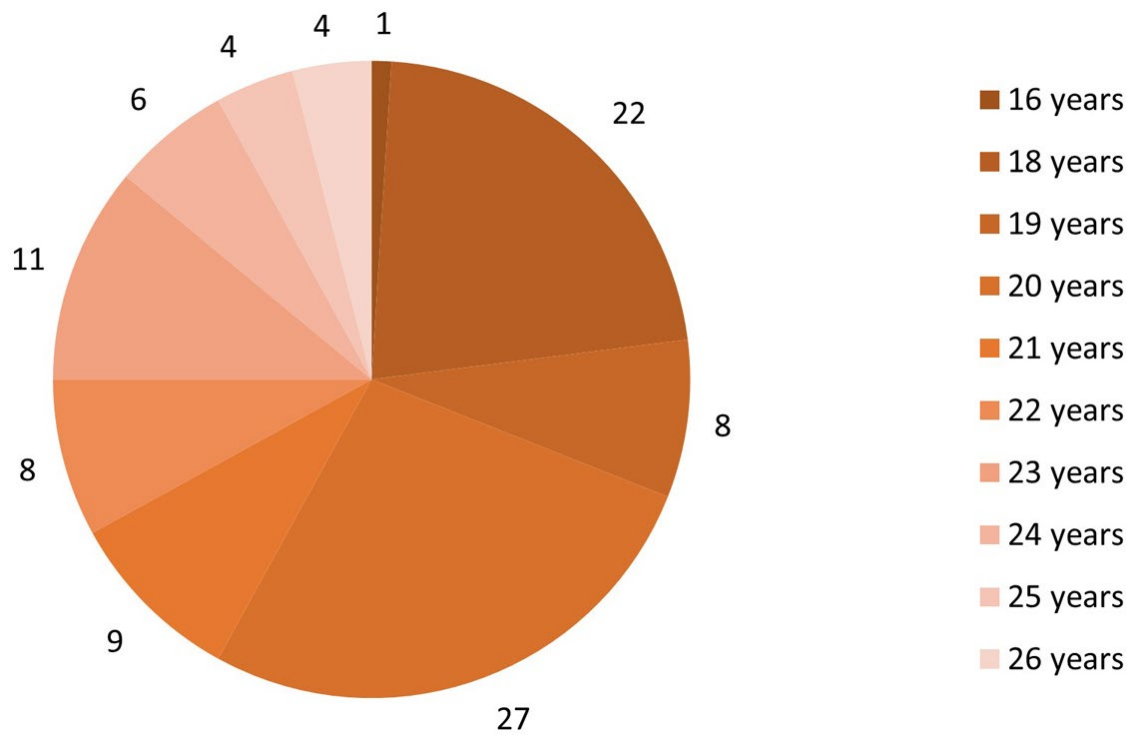
Distribution of respondents by age.

In total, 150 respondents participated in the research survey. The return rate of the questionnaire was 88%, or 132 returned samples. Based on incorrect or incomplete filling in, some samples had to be discarded; out of the total 132 returned samples, 106 samples remained for use. Due to large age differences and some sports sectors being more predominant, further samples were subsequently discarded. Therefore, the total number of questionnaires used equals 100 samples. Of the 100 available samples, 79 women and 21 men (mostly bodybuilders—13 bodybuilding, four gymnastics, and four dance) participated in the research investigation. Furthermore, out of the 100 available samples, 20 samples were from each sports sector. The age limit of the respondents ranged from 16 to 26 years. The arithmetic mean was 20.7 years, modus and median 20 years, standard deviation 2.33 (Figure 1).

## 4. Methods

In general, the EAT test focuses on three main factors: dieting behavior, bulimia, and increased interest in food and oral control. The utilized version of the test contains 26 closed questions with a choice of 6 scale answers (always, very often, often, sometimes, rarely, and never).

There was an analysis of individual test questions and their possible modification according to the difference between the general population and athletes (functional sports nutrition is characterized primarily by the control of energy intake, nutrients intake, their timing in relation to training and, with it, great demands on self-control, as well as control of body weight and body composition).

Question No. 1 *I am afraid of being overweight* was rephrased as *I am afraid of gaining body weight despite my strict diet and training regime.*

Question No. 2 *I avoid eating when I’m hungry* was eliminated.

Question No. 3 *I think of myself as worrying too much about food* was changed to *I feel anxious if I am not in control of my diet regimen.*

Question No. 4 *It happens that I start to overeat, and I feel like I will not be able to stop* was changed and questions were created to determine the athlete’s relationship to overeating *In my strict regime, I treat myself to one “cheat day,” When I have a “cheat day” or I am on an “off season mode,” I eat, even when I am no longer hungry or do not have appetite. After a “cheat day” or after eating something that is not on my diet plan, I feel sick and have a guilty conscience.*

Question No. 5 *I cut food into small pieces* was eliminated.

Question No. 6 *I am aware of the caloric value of the food I eat* was kept and enriched with other questions determining the degree of seriousness of awareness of the caloric value of meals: *I am aware of the caloric value of the food I eat, I count my calorie intake, I feel anxious if I do not have an overview of the caloric values of the food I eat, I do not eat any food whose caloric value I do not know and does not fit my caloric intake, When I eat my daily caloric intake, I’m usually resigned to not eating anything again*.

Question No. 7 *I often avoid foods high in carbohydrates (bread, potatoes, rice,* etc.*)* was edited and more questions were added: *Avoiding foods with a high carbohydrate content (bread, potatoes, rice,* etc.*) is part of my diet plan, When I eat a meal with a high content of carbohydrates, I am afraid of gaining body weight.*

Question No. 8 *I feel that others would like me to eat more* was eliminated.

Question No. 9 *I vomit after eating* was eliminated.

Question No. 10 *I feel very guilty after eating* was excluded from the questionnaire due to its inapplicability to the group of athletes.

Question No. 11 *I think too often about wanting to be slimmer* has been changed and additional questions were added to determine the athlete’s relationship to their energy intake: *I feel that my diet regime and training are insufficient and I would like to be slimmer, Despite my special diet and training regimen, I deliberately reduce energy intake to be slimmer, I want to be slimmer and thus improve my athletic performance, I want to be slimmer and thus improve my physical appearance, I do not care about sports performance.*

Question No. 12 *When I exercise, I think about how many calories I burn* was changed and additional questions were added to determine the relationship between the individual’s feelings and exercise: *If I eat something outside of my meal plan, I usually add an extra workout, I regularly add extra training or exercises, After mastering the training, I feel stronger and more self-confident, If I have to miss my workout, I feel anxious, If I had to choose between hanging out with friends/family and training, I would choose training, Only through training will I get rid of a bad mood and forget about my problems, I exercise when I am sick or injured.*

Question No. 13 *People think I’m too thin* was changed and additional questions regarding the relationship between the coach’s opinion and the athlete’s behavior were added: *My coach thinks I should be slimmer, My coach’s opinion affects my eating behavior.*

Question No. 14 *I keep thinking that I have a lot of body fat* was changed and additional questions were added to determine the basic relationship of an individual to their body, regardless of sports performance: *I feel good in my body, I feel good about my physical “self,” I like my body, I nurture and take care of it.*

Question No. 15 *It takes longer for me to finish my food than for others* was excluded.

Question No. 16 *I avoid foods with sugar content* was reformulated in the same manner as question No. 7 and additional questions were added that could potentially capture any pathologies present: *Avoiding foods with sugar content is part of my diet plan, My meal plan allows for foods with sugar content once in a while, I do not eat any food that contains sugar, even though my meal plan allows it, When I eat food that contains sugar, I am afraid of gaining body weight.*

Question No. 17 *I eat diet foods* was excluded from the questionnaire due to its inapplicability to the group of athletes.

Question No. 18 *I feel like food is controlling my life* was excluded.

Question No. 19 *I exercise self-control when it comes to food* was excluded.

Question No. 20 *I feel that others are forcing me to eat* was excluded from the questionnaire due to its inapplicability to the group of athletes.

Question No. 21 *I spend too much time thinking about food* was excluded and replaced by those mentioned above in question No. 3.

Question No. 22 *When I eat sweets, I feel uncomfortable* was amended and additional questions were added regarding the individual’s relationship to the consumption of healthy foods: *When I eat something with less healthy ingredients, I feel uncomfortable, I never eat anything if I do not know the exact composition, preparation method, or origin of the food, In principle I prefer to prepare my food by myself, I study in detail the composition and origin of the food on the back of the packaging, When I choose my food, its quality is more important to me than its taste, My diet regimen consists of a rich variety of foods and ingredients.*

Question No. 23 *I follow diets* was excluded from the questionnaire due to its inapplicability to the group of athletes.

Question No. 24 *I like the feeling of an empty stomach* was excluded.

Question No. 25 *After eating, I have the urge to vomit* was excluded.

Question No. 26 *I like to try new substantial foods* was excluded.

From the original 26 questions, a diagnostic tool of 38 questions came into existence. The evaluation for the new tool for detecting pathological forms of eating behavior was adjusted to only a four-point scale: always, often, rarely, and never.

The evaluation was determined in the following manner:

In the questions: 1, 2, 3, 6, 7, 8, 9, 10, 11, 13, 14, 15, 17, 18, 20, 21, 23, 24, 25, 26, 27, 28, 29, 30, 31, 32, 33, 35, 36, 37

- Always = 3 points
- Often = 2 points
- Rarely = 1 point
- Never = 0 points

In the questions: 4, 5, 12, 16, 19, 22, 34, 38

- Always = 0 points
- Often = 1 point
- Rarely = 2 points
- Never = 3 points

The evaluation of the newly created instrument is based on the same principle as the evaluation of the EAT-26 test. Each answer is assigned a given number of points, after adding them up we get a total score. The cut-off score limit was set at 57 points, i.e., exactly half of the possible points obtained. We followed the evaluation of the EAT-26, where the cut-off score limit was set in the same way. A score of 57 and above may indicate disturbed eating behavior and a potential risk of developing an eating disorder (Table 2).

**Table 2:** Newly created questionnaire.

I am afraid of gaining weight despite my strict diet and training regime.	Always	Often	Rarely	Never
I am aware of the caloric value of the food I eat.	Always	Often	Rarely	Never
If I eat something outside of my meal plan, I usually add an extra workout.	Always	Often	Rarely	Never
I feel comfortable in my body.	Always	Often	Rarely	Never
In my strict regime, I treat myself to 1 day of a “cheat day.”	Always	Often	Rarely	Never
In general, I prefer to prepare my food by myself.	Always	Often	Rarely	Never
I want to be slimmer and thus improve my sports performance.	Always	Often	Rarely	Never
I am always counting my calorie intake.	Always	Often	Rarely	Never
Avoiding foods with a high carbohydrate content (bread, potatoes, rice, etc.) is part of my diet plan.	Always	Often	Rarely	Never
I regularly add extra training or exercises.	Always	Often	Rarely	Never
I feel anxious if I am not in control of my diet regimen.	Always	Often	Rarely	Never
I want to be slimmer and thus improve my physical appearance, I do not care about my sports performance.	Always	Often	Rarely	Never
Avoiding foods with sugar content is part of my diet plan.	Always	Often	Rarely	Never
My coach thinks I should be slimmer.	Always	Often	Rarely	Never
I study in detail the composition and origin of the food on the back of the packaging.	Always	Often	Rarely	Never
After mastering the training, I feel stronger and more self-confident.	Always	Often	Rarely	Never
When I eat a meal with a high content of carbohydrates, I am afraid of gaining body weight.	Always	Often	Rarely	Never
When I have a “cheat day” or I am on an “off season mode,” I eat, even when I am no longer hungry or do not have appetite.	Always	Often	Rarely	Never
I feel good about my physical “self.”	Always	Often	Rarely	Never
When I choose my food, its quality is more important to me than its taste.	Always	Often	Rarely	Never
If I have to miss my workout, I feel anxious.	Always	Often	Rarely	Never
My meal plan allows for foods with sugar content once in a while.	Always	Often	Rarely	Never
My coach’s opinion affects me in my eating behavior.	Always	Often	Rarely	Never
After a “cheat day” or after eating something that is not on my diet plan, I feel sick and have a guilty conscience.	Always	Often	Rarely	Never
If I have to choose between hanging out with friends/family and training, I would choose training.	Always	Often	Rarely	Never
I feel anxious if I do not have an overview of the caloric values of the food I eat.	Always	Often	Rarely	Never
I do not eat any food that contains sugar, even though my meal plan allows it.	Always	Often	Rarely	Never
I feel that my diet regime and training are insufficient, and I would like to be slimmer.	Always	Often	Rarely	Never
Only through training will I get rid of a bad mood and forget about my problems.	Always	Often	Rarely	Never
I do not eat any food whose caloric value I do not know and does not fit my caloric intake.	Always	Often	Rarely	Never
When I eat something with less healthy ingredients, I feel uncomfortable.	Always	Often	Rarely	Never
Despite my special diet and training regimen, I deliberately reduce my energy intake to be slimmer.	Always	Often	Rarely	Never
When I eat my daily caloric intake, I’m usually resigned to not eating anything again.	Always	Often	Rarely	Never
I like my body; I nurture and take care of it.	Always	Often	Rarely	Never
When I eat a meal with a high content of carbohydrates, I am afraid of gaining body weight.	Always	Often	Rarely	Never
I never eat anything if I do not know the exact composition, preparation method or origin of the food.	Always	Often	Rarely	Never
I exercise when I am sick or injured.	Always	Often	Rarely	Never
My diet regimen consists of a rich variety of foods and ingredients.	Always	Often	Rarely	Never

Exploratory factor analysis method was used for data processing (we did not assume that the factors would remain the same as for EAT-26). Data were processed using SPSS 26 software.

## 5. Results

Exploratory factor analysis of the newly created questionnaire items led to the following results.

Two questions were excluded during data processing: question No. 14—“*I feel good in my body.*” and question No. 19—“*I feel good about my physical self.*” They showed a completely illogical reversal of the correlation. The cause of this fact may be a poor understanding of the questions by the respondents, or a poor formulation of both questions during the creation of the questionnaire.

Factor analysis among the questionnaire questions found five strong common significant factors, which were:

- Control over diet
- Control over body weight
- An obsession with training
- Appetite for food
- Calculating caloric intake

**Figure 2:**
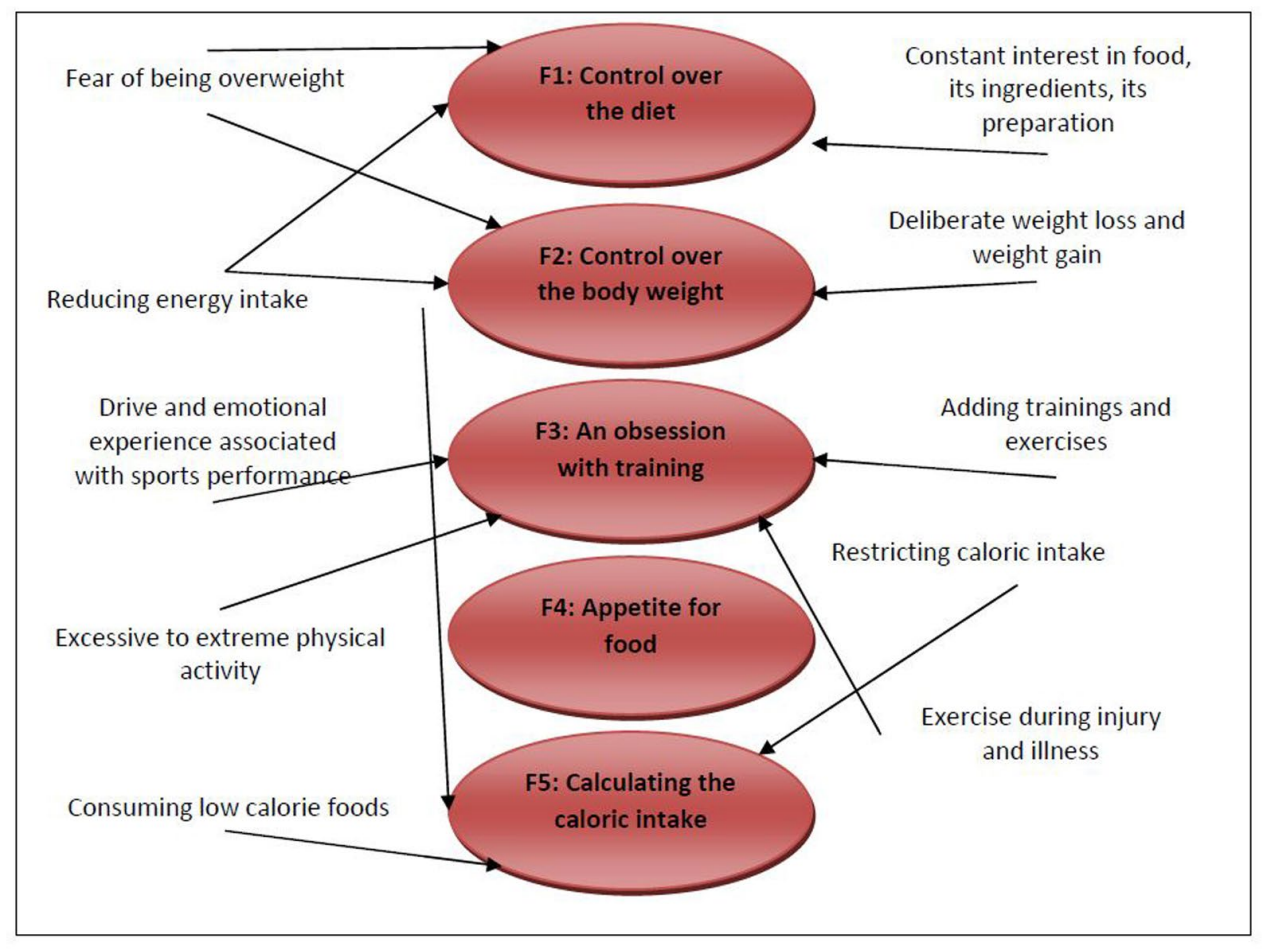
Classification of some pathological features under the discovered factors.

The factors found are very closely related to the characteristic features of pathological eating behavior in athletes (Figure 2).

Factor F1 included 15 questions, factor F2 included seven questions, factor F3 included five questions, factor F4 included five questions, and factor F5 included three questions (Table 3).

**Table 3:** Exploratory factor analysis results.

**Question**	**F1**	**F2**	**F3**	**F4**	**F5**
I am afraid of gaining weight despite my strict diet and training regime.	−0.0285	**0.67516**	0.07253	0.0302	0.08335
I am aware of the caloric value of the food I eat.	**0.42261**	0.08845	0.18977	−0.1378	**0.36068**
If I eat something outside of my meal plan, I usually add an extra workout.	0.12354	0.0018	**0.61977**	0.09148	0.32839
In my strict regime, I treat myself to 1 day of a “cheat day.”	−0.178	0.05764	0.16755	**0.35833**	0.00787
In general, I prefer to prepare my food by myself.	0.31664	−0.1254	0.15758	−0.2366	0.30822
I want to be slimmer and thus improve my sports performance.	−0.0761	**0.60765**	0.07578	−0.0588	0.01691
I am always counting my calorie intake.	**0.64173**	−0.176	0.16467	0.0889	**0.61676**
Avoiding foods with a high carbohydrate content (bread, potatoes, rice, etc.) is part of my diet plan.	**0.62001**	0.14901	−0.0679	−0.0382	0.1925
I regularly add extra training or exercises.	−0.032	−0.0347	**0.74655**	0.00968	0.27469
I feel anxious if I am not in control of my diet regimen.	**0.61546**	0.19554	0.0805	−0.1486	0.18338
I want to be slimmer and thus improve my physical appearance, I don't care about my sports performance.	0.18771	−0.2819	0.07018	0.04416	−0.0966
Avoiding foods with sugar content is part of my diet plan.	**0.69235**	0.16379	−0.1851	−0.1213	0.20364
My coach thinks I should be slimmer.	−0.0893	**0.432**	0.16659	**0.37645**	0.20082
I study in detail the composition and origin of the food on the back of the packaging.	**0.78773**	−0.2012	0.10199	−0.0407	0.05464
After mastering the training, I feel stronger and more self-confident.	0.05875	0.15059	−0.6223	0.3349	0.16131
When I eat a meal with a high content of carbohydrates, I am afraid of gaining body weight.	0.17791	**0.57765**	0.12283	−0.2976	−0.0284
When I have a “cheat day” or I am on an “off season mode,” I eat, even when I am no longer hungry or don’t have appetite.	0.04132	0.18151	0.10764	−0.6029	0.12464
When I choose my food, its quality is more important to me than its taste.	**0.70602**	−0.2601	0.02179	0.00464	−0.0054
If I have to miss my workout, I feel anxious.	0.26869	−0.0118	0.31489	0.13638	−0.1108
My meal plan allows for foods with sugar content once in a while.	0.02571	−0.2285	−0.0895	**0.71044**	0.05346
My coach's opinion affects me in my eating behaviour.	0.26281	0.33764	0.32878	0.19397	0.01947
After a “cheat day” or after eating something that is not on my diet plan, I feel sick and have a guilty conscience.	0.3245	**0.4673**	0.05311	−0.1339	−0.0145
If I have to choose between hanging out with friends/family and training, I would choose training.	−0.2111	0.17431	**0.70636**	0.15571	−0.0405
I feel anxious if I don’t have an overview of the caloric values of the food I eat.	**0.76682**	0.09958	0.03205	−0.0477	0.24645
I don’t eat any food that contains sugar, even though my meal plan allows it.	**0.71965**	0.03306	−0.0855	0.21787	−0.1669
I feel that my diet regime and training are insufficient, and I would like to be slimmer.	0.07346	**0.67515**	0.04247	−0.0798	−0.2552
Only through training will I get rid of a bad mood and forget about my problems.	−0.0994	0.02593	**0.35571**	−0.15	0.02581
I do not eat any food whose caloric value I do not know and does not fit my caloric intake.	**0.95453**	−0.191	−0.0467	0.1036	0.21382
When I eat something with less healthy ingredients, I feel uncomfortable.	**0.43364**	0.14112	−0.0176	**0.43456**	−0.1075
Despite my special diet and training regimen, I deliberately reduce my energy intake to be slimmer.	**0.51999**	0.26496	0.00764	−0.1922	−0.1842
When I eat my daily caloric intake, I’m usually resigned to not eating anything again.	**0.85938**	0.03889	−0.0914	−0.2136	0.01943
I like my body; I nurture and take care of it.	0.15525	0.28888	−0.0567	**0.48155**	0.0718
When I eat a meal with a high content of carbohydrates, I am afraid of gaining body weight.	0.20255	**0.61078**	−0.2361	0.09558	−0.3003
I never eat anything if I don't know the exact composition, preparation method or origin of the food.	**0.77575**	0.0326	−0.0967	0.02187	0.01445
I exercise when I am sick or injured.	0.11773	0.11263	**0.51471**	0.06772	−0.0263
My diet regimen consists of a rich variety of foods and ingredients.	**0.69025**	0.09148	−0.1946	0.20043	**0.42186**

Bold values means the correlation coefficients for selected questions grouped under each factor.

Using the discovered factors, new variables can be created that can be used in further research instead of the original items. When focusing in detail on the original items of the questionnaire, it is possible to observe which questions are grouped under which factor. Important questions related to awareness of the caloric value of food, avoiding individual nutritional components, monitoring the composition of individual foods, etc. are grouped under the Control over the diet factor. Respondents with this factor responded very positively to the question *“I do not eat any food the caloric value of which I do not know, and which does not fit my caloric intake.,”* or to the question *“When I eat my daily caloric intake, I’m usually resigned to not eating anymore..”* Significantly frequent were the questions related to monitoring the composition and origin of foods on their packaging, feeling anxious when there is insufficient insight into the caloric values of food and, the compulsive need to know all the information about the food consumed. It can be said that *Control over the diet* defines all the common signs appearing in athletes with disturbed eating behavior.

*Control over body weight*, a second factor, was found, and under this factor are grouped the questions related mainly to the fear of weight gain and the inner feeling concerning being thinner. Respondents with this factor responded very positively to the question *“I am afraid of gaining body weight despite my strict diet and training regime.,”* and to the question “*I feel that my diet regime and training are insufficient and I would like to be slimmer..”* Furthermore, the respondents reacted very positively to the question “*I want to be slimmer and thus improve my athletic performance..”* This testifies to the aforementioned fact that athletes, in contrast to non-athletes, for whom slimness is often the only goal, associate it with sports performance. So, they take a point of view imitating the equation: “I want to improve my sports performance = I have to lose weight.” This fact is manifested when comparing the mutual correlation of the factor and the relevant questions, specifically the question already mentioned above “*I want to be slimmer and thus improve my athletic performance.,”* and the question “*I want to be slimmer and thus improve my physical appearance, I do not care about sports performance..”* The first question concerning sports performance is highly correlated with the factor at 0.60765, while the second question concerning physical appearance shows a completely insignificant negative value of −0.2813 of correlation with the factor. Even in this case, it can be said that the signs of behavior grouped under the factor occur commonly in athletes, and the factor was chosen correctly.

The third factor was named *An Obsession with Training*. Questions related mainly to training are grouped under this factor. Respondents responded very positively to the questions *“I regularly add extra training.”* and *“If I have to choose between hanging out with friends/family and training, I will choose training..”* Even in this case, it is clear that the factor reflects common signs of disturbed eating behavior in athletes. Not only do athletes evaluate their body weight through athletic performance, but they also subsequently change their training, which is aimed at athletic performance. They add training, exercise when injured or sick, express all their emotions through training, and make training a priority.

As a fourth factor, we found a factor we named *Appetite for Food*. Under this factor, important questions related to how the athlete manipulates their eating regimen were grouped. For many athletes with disordered eating behaviors, a strict and restrictive eating regimen can be an advantage behind which they can hide their true concerns about their bodies. The second option are athletes who do not have as strict an eating regimen set in the first case, but they still avoid various foods and meals. And it is under this factor that such questions are grouped, and the questions relate to what the athlete’s eating regimen allows and whether they are committing a so-called sin, specifically, for example, the question *“In my strict regime, I treat myself to one ‘cheat day.’”* Unpleasant feelings after eating, feelings of guilt, and subsequent compensatory behavior are typical of bulimic behavior. Athletes may have a tendency to escape to certain types of food, given that they cannot indulge in them in their strict diet. However, after eating these foods, they subsequently feel guilty, because they violated their eating regimen, and subsequently a chain of typical behaviors and characteristic emotions (guilt, feeling guilty, aggression, etc.) is triggered. Also grouped under this factor are questions regarding the coach’s mindset with regard to the athlete’s physical appearance and the athlete’s own mindset in conjunction with their body. In short, the factor describes how the athlete approaches their eating regimen.

The last, fifth factor, was a factor named *Calculating the Caloric Intake*. Under this factor, as the name suggests, questions related to caloric intake calculation and awareness of the caloric value of food are grouped. Calculating caloric intake and knowing caloric values is not at all unusual for competitive athletes, many athletes even create their own meal plan and thus they have to work with individual values. However, what is important is to what extent it is the norm and when counting caloric intake can become a pathology.

### 5.1. Point score of the respondents

As already mentioned above, compared to the original EAT-26 questionnaire, the point score was adjusted, and its cut-off value was set at 57 points and above. Of the respondents, 33%, i.e., 33 out of a possible 100, achieved the specified or higher value.

Respondents with a point score of 57 and above were found in every sport tested. Of the 33 respondents reaching the point limit, 6% of respondents were in aerobics, 24% of respondents in gymnastics, 15% of respondents in professional dance, 27% of respondents in figure skating, and 27% of respondents were among bodybuilding/fitness. The largest number of respondents with a point score of 57 and above was found in figure skating and bodybuilding/fitness, where nine respondents achieved a point score in both sports. The least was found in aerobics, where only two respondents achieved the point score. Among all 33 respondents scoring 57 and above, there were seven males and 26 females, with all seven males engaged in the same sport, bodybuilding/fitness.

From the group of respondents achieving a point score of 57 or higher, the youngest respondent was 18 years old, while the oldest was 26 years old. The rest of the respondents achieving a point score of 57 and higher were most often between 20 and 23 years old, which can be characterized as a typical age for a peak career in most aesthetic sports.

### 5.2. Point score by sport branch

Subsequently, individual scores were also compared according to the sports sector. One respondent could get a maximum number of points 108. So, all 20 respondents from the same sport could reach a limit of 2,160 points. In total, all respondents could get a total of 10,800 points. The total number of points of all respondents reached roughly half of the possible points obtained; all respondents together obtained 5,026 points. Of these, the largest number of points was obtained by respondents from bodybuilding and fitness, who together obtained 1,175 points. Bodybuilding and fitness are very specific because of their diet regimens. Athletes often go through different phases of their diets and their preparations, shock the body by starving or, on the contrary, by overeating, and last but not least, more than anyone else, they adhere to a strict and exact diet plan. There exist several studies that describe the occurrence of eating disorders in this specific sport branch, for example, anorexia nervosa is often described in female competitors in the category of bikini fitness. The next highest possible number of points obtained belonged to gymnastics and figure skating, where the respondents from a gymnastics background received a combined 1,069 points, and the respondents from a figure skating background received a combined 1,006 points. Both sports are very characteristic for their technical elements, where lower body weight is a great advantage. Athletes very often reduce their body weight in order to include the most complicated technical elements in their sets. Gymnastics is also a very common sport, where the occurrence of eating disorders has already been described several times. Subsequently, aerobics was ranked after gymnastics and figure skating, where all respondents together scored 951 points. Aerobics can be very similar to gymnastics in some ways and is also very typical for its technical elements where a lower body weight can be an advantage. At the same time, athletes are evaluated by so-called artistic judges, who evaluate the athlete’s appearance and demeanor. The least number of points was awarded to dancing, where all respondents together scored 825 points. The difference between bodybuilding/fitness and dancing is very interesting, where the difference is up to 42% (Table 4).

**Table 4:** Number of points achieved in individual sports branches.

Sport branch	Number of points	Number of respondents	The average number of points
Bodybuilding/fitness	1,175	20	59
Gymnastics	1,069	20	53
Figure skating	1,006	20	50
Aerobics	951	20	48
Dancing	825	20	41

Understandably, respondents from the bodybuilding and fitness sports branches scored the most points on average, as they were the only ones who exceeded the threshold of 57 points on average. Respondents from the gymnastics, figure skating, and aerobics industries stayed just below the threshold of 57 points. The respondents from the dancing area did the best in the average number of points. From the researched group, athletes from the bodybuilding and fitness sports sectors had a much more frequent tendency to exceed the specified point limit and it can be said that they have a much greater tendency toward disordered eating behavior than other respondents from other sports sectors. However, it is not possible to carry out a detailed analysis of all variables that would show which of the sports mentioned above has the greatest tendency to the occurrence of disturbed eating behavior or eating disorders, due to the small number of respondents in each sports group.

Among all 33 respondents scoring 57 and above, there were seven males and 26 females, with all seven males involved in the same sport, namely bodybuilding/fitness.

## 6. Discussion

Factor analysis discovered five common significant factors: Control over the diet, Control over the body weight, An obsession with training, Appetite for food, and Calculating the caloric intake. All the factors found can be considered common characteristics for the eating and training regimen of athletes, as well as areas that can be transferred to the level of pathological behavior. It is therefore necessary to respect these factors as part of the training process, but at the same time take their degree into consideration.

de Fortes et al. (2016) verified in their study the psychometric properties of the Disordered Eating in Sports Scale (DES) created by them using 1,338 Brazilian athletes and compared it with the EAT-26 test. They also state that the EAT-26 test does not respect the behavior and needs of the athletes. Doninger et al. (2005) verified the psychometric properties of the EAT-26 test in athletes on a group of 207 female athletes. The factor analysis resulted in five factors: Drive for Thinness, Food Preoccupation, Others’ Perceptions, Purging Behavior, and Dieting Behavior. These factors have some elements in common with the factors found in our study.

Martinsen et al. (2010) examined eating behavior in adolescent elite athletes and compared it to a non-athlete control group. Their study entitled *“Dieting to win or to be thin?”* describes dietary behavior as the most important factor in athletes. The authors of the study consider diets and dietary behavior to be the most typical feature of elite athletes, and at the same time describe a very thin line between dietary behavior used to improve sports performance and eventual success, and dietary behavior that can very easily turn into a pathological level. At the same time, this study confirms the appropriateness of the selection of some questionnaire questions regarding dietary behavior. For example, one of the many questions might be a system of questions: “*Avoiding foods with sugar content is part of my diet plan,” “My meal plan allows for foods with sugar content once in a while,” “I do not eat any food that contains sugar, even though my meal plan allows it.”* These example questions clearly highlight the fact mentioned above; the first question is aimed at mapping the athlete’s eating regimen, the second question is simultaneously based on the first question, however it expands the evaluator’s overview of how strict the athlete’s eating regimen is, and finally the third and last question can indicate whether the eating behavior is a norm or a pathology. For example, 58 respondents answered *“never”* to the last question regarding whether or not an athlete allows himself to eat food containing sugar. These results continue to confirm the claim that the overall point score should only serve as an informative one and the individual answers of the respondents should subsequently be analyzed in detail despite the fact that the respondent did not reach the given point score limit. In other words, “only” 33 respondents reached the threshold of 57 points and above, but as can be seen, far more respondents avoid sugary foods, even if they do not have to. Another study confirming the factors representing part of the training process and risk was carried out by Francisco et al. (2012) in aesthetic sportswomen, specifically in elite dancers and gymnasts. The study dealt with the investigation of specific characteristics in the dance and gymnastics environment. The results of the study showed that the aforementioned characteristics are also risk factors for the emergence of disturbed eating behavior and the development of eating disorders. Specifically, the results showed that 58% of elite dancers and gymnasts exhibited risk factors, while in 29% these were sport-specific. This fact underlines not only the results of this study, when the characteristic features of sport are also referred to as risk factors, but also the risk context of all aesthetic sports. However, the question remains how to prevent the tendency to follow the rules of the given sports from turning into a risk of pathological behavior. From the theoretical findings, it follows that the biggest influence is attributed to the coaches, the increasing demands of the given sport, and the competition. In other words, facts that are difficult to eliminate. However, it is possible to intervene in the area of the athlete’s perception of these influences and increase his resistance to them, which is implemented within the framework of an individual approach to each athlete and close cooperation between a coach and a sports psychologist.

Sundgot-Borgen and Garthe (2011) consider the extreme methods of weight reduction used in sports with weight categories to be risky for the emergence of pathological eating behavior and describe preventive strategies that could prevent the current situation, including the development of sports education programs for coaches and athletes themselves and adjustments to sports rules in some sports.

There are also suggestions by researchers that modern technologies, including apps aimed at controlling energy intake and monitoring physical activity, may promote the development of pathological eating behavior, as confirmed by studies such as Simpson and Mazzeo (2017). In contrast, the study by Hahn et al. (2021) did not confirm this.

Piepiora et al. (2018) looked at the relationship between body composition and eating behavior in female wrestlers, but no relationship was found in their study. Piepiora et al. (2017) also investigated the relationship between personality characteristics as measured by the NEO-FFI and eating behavior in young female wrestlers and tennis and volleyball players, but no relationship was found.

The coach’s approach to their client therefore plays a significant role. In many cases, the coach is the closest person to the athlete, with whom the athlete often spends the most time. The coach’s influence is also indicated by the answers to the question *“Does my coach’s opinion influence me in my eating behavior.”* in our study, where only 11 respondents gave a completely negative answer of “never.” More than half of the respondents answered “always” (23 respondents) and “often” (32 respondents), the neutral answer “rarely” was subsequently chosen by 34 respondents. In some cases of more sensitive and introverted individuals, the coach’s attitude and opinion can be the decisive factor in the emergence of pathological eating behavior.

The main results of this research investigation therefore brought positive findings, namely that the newly created questionnaire is applicable to a group of competitive athletes and can fully reflect the common characteristics of an athlete’s regimen. However, it does not provide a solution to the problem that lies in the coaching team’s approach to the athlete and in the athlete’s behavior under the action of environmental influences. It is important to ensure high-quality and professional conditions for athletes, an individual approach to the athlete, and full care in all spheres of sports preparation (training care, psychological care, and health care).

The weakness of this and other verbal methods is the distortion of respondents’ answers, when many of them associate a truthful answer with the fear of confessing to pathological eating behavior. Therefore, respondents subsequently choose the answers they think are correct, regardless of whether they have or perceive a problem in that area. Defense mechanisms also play a role; typically it is rationalization, when the athlete unintentionally answers the questionnaire differently to how they behaves in reality.

The phenomenon of pathological eating behavior in sports does not have a definitive solution, but for professionals working in this area, it is an important task to try to act preventively and to solve any pathological conditions that may arise as soon as possible in order to preserve the health of the athlete. It is also possible that they may encounter an ethical dilemma, where they will have to balance the current success of the athlete against their health.

## 7. Conclusion

The EAT-26 questionnaire was first analyzed in detail and reworked into a form that could be applied to a group of competitive athletes. The main goal of the study was to confirm the correctness and appropriateness of the newly chosen questions. Factor analysis discovered five strong common factors that replicate characteristics that athletes are very familiar with from their sports training. In addition to the factor analysis, an analysis of the respondents’ point scores was subsequently performed. The amount of the point score helps to determine whether the individual may have disturbed eating behavior or an eating disorder. However, the point score should only serve as a guideline, and attention should also be paid to individuals who did not reach the specified point score limit. Eating disorders are a very intimate topic and their diagnosis can be very difficult. Diagnostics in the field of performance sports can then be particularly demanding. Therefore, even the most banal symptoms should be paid attention to. The athlete’s body and his physical and mental health represent an important key to success. It should also be approached in such a spirit, not as a human machine. We plan to follow up on our research with further studies using a larger number of respondents, or the utilization of the questionnaire in other sports groups as well, not only in aesthetic sports. This is a pilot study representing the first step in the overall validation process of the new method.

## Data availability statement

The original contributions presented in the study are included in the article/supplementary material, further inquiries can be directed to the corresponding author.

## Ethics statement

The studies involving human participants were reviewed and approved by the Ethic Committee of Palestra. Written informed consent to participate in this study was provided by the participants’ legal guardian/next of kin.

## Author contributions

DS and TB conceived and designed the research. TB performed the research and analyzed the data. DS interpreted the results. BP drafted and edited manuscript. All authors critically revised manuscript and approved the final version of the manuscript.

## Conflict of interest

The authors declare that the research was conducted in the absence of any commercial or financial relationships that could be construed as a potential conflict of interest.

## Publisher’s note

All claims expressed in this article are solely those of the authors and do not necessarily represent those of their affiliated organizations, or those of the publisher, the editors and the reviewers. Any product that may be evaluated in this article, or claim that may be made by its manufacturer, is not guaranteed or endorsed by the publisher.
